# Healing Hanuman’s Army: Veterinary Care as a Core Component of One Health Principles in a Southeast Asian Monkey Forest

**DOI:** 10.3390/ani14010117

**Published:** 2023-12-28

**Authors:** James E. Loudon, Michaela E. Howells, Christopher A. Wolfe, I. Nyoman Buana, Wayan Buda, I. Nengah Wandia, I. Gusti Agung Arta Putra, Meghan Patterson, Agustín Fuentes

**Affiliations:** 1 Department of Anthropology, East Carolina University, Greenville, NC 27858, USA; wolfec23@ecu.edu; 2 Department of Anthropology, University of North Carolina-Wilmington, Wilmington, NC 28403, USA; howellsm@uncw.edu (M.E.H.);; 3 Ubud Monkey Forest, Padangtegal 80571, Bali, Indonesia; 4 Primate Division of Natural Resources and Environment Research Center, Universitas Udayana, Denpasar 80361, Bali, Indonesia; wandia@unud.ac.id (I.N.W.); artaputra@unud.ac.id (I.G.A.A.P.); 5 Department of Anthropology, Princeton University, Princeton, NJ 08544, USA; afuentes2@princeton.edu

**Keywords:** veterinary care, injury, mortality, human–alloprimate interplays, One Health, anthropogenically disturbed habitats, Ubud Monkey Forest, Padangtegal, long-tailed macaques, *Macaca fascicularis*

## Abstract

**Simple Summary:**

This study examines patterns of injury and mortality in long-tailed macaques (*Macaca fascicularis*) at the Ubud Monkey Forest in Bali, Indonesia. This forest is situated in an urban landscape that is continuously developed and modified to suit the needs of international tourism. The macaques at the site face the typical challenges of living in social groups, and novel challenges of negotiating an urban environment. Forest management has actively committed to the physical and psychological health of the macaques for over 40 years. The addition of a veterinary clinic in the last decade addresses the needs of injured macaques, while inadvertently strengthening their One Health framework. In this collaborative work, we review patterns of injury and mortality among individuals brought to the clinic over a four-year span. Males and younger macaques were over-represented among injured individuals seen at the veterinary clinic. The most common natural causes of harm were from falls and macaque-inflicted wounds. The most common anthropogenic causes of harm were vehicular collisions, electrocutions, gunshots, and plastic pollution. These results are critical for effectively managing wildlife in urban areas across the globe. This study demonstrates the importance of local stakeholders’ knowledge of their environment and effective programs to manage wildlife by synthesizing conventional conservation initiatives with cultural patterns, and religious and philosophical approaches.

**Abstract:**

Wildlife that inhabit urban landscapes face the dual challenge of negotiating their positions in their group while navigating obstacles of anthropogenically modified landscapes. The dynamics of urban environments can result in novel injuries and mortalities for these animals. However, these negative impacts can be mitigated through planning, and onsite veterinary care like that provided by the Ubud Monkey Forest in Bali, Indonesia. We examined 275 recorded injuries and mortalities among six social groups of long-tailed macaques (*Macaca fascicularis*) brought to the veterinary clinic from 2015–2018. We fit the probabilities of injury vs. death among macaques brought to the clinic using a multilevel logistic regression model to infer the relationship between injury vs. death and associated demographic parameters. Males were more likely to sustain injuries and females were more likely to die. The frequency of injuries and mortalities changed over the four-year study period, which was reflected in our model. The odds of mortality were highest among young macaques and the odds of injury vs. mortality varied across the six social groups. We categorized injuries and mortalities as “natural” or “anthropogenic”. Most injuries and mortalities were naturally occurring, but powerlines, motorized vehicles, and plastic present ongoing anthropogenic threats to macaque health. Most wounds and injuries were successfully treated, with healthy animals released back to their group. We suggest other sites with high levels of human–alloprimate interplays consider the Ubud Monkey Forest veterinary office as a model of care and potentially adopt their approaches.

## 1. Introduction

As human populations continue to grow and expand, the overlap with wildlife has increased. Animals inhabiting cities, urban landscapes, and anthropogenically disturbed habitats frequently rely on behavioral flexibility and their ability to employ behavioral modifications to exist in ecosystems with artificial infrastructures [1]. Urban ecologists have documented a number of avian and mammalian species who can readily adapt to anthropogenically modified environments and exist alongside humans [2]. In some of the zones of human–alloprimate sympatry in Africa, Asia, and the Caribbean, Old World monkeys and humans are intimately interconnected [3]. Throughout southeast Asia, humans and macaques (*Macaca* spp.) have lived among each other for millennia. Macaques are among the most successful genus of alloprimates. Their wide geographic distribution is linked to their high degrees of behavioral plasticity, cognitive complexity, and ability to live in a variety of habitats, including anthropogenically disturbed landscapes and urban centers [4,5]. These traits and their ability to exploit anthropogenically disturbed habitats has led to the human–macaque interface.

In Bali, Indonesia, there has been a shift towards urbanization and habitat conversion resulting in more land dedicated to infrastructure for international tourism. As a result, there is less land available for traditional uses including terraced rice agriculture (*sawah*) [6]. Increased investment in the tourism economy has resulted in the protection of forest fragments across the island. Some forest fragments are associated with sacred Hindu temple complexes, which provide habitat for an economically and culturally important alloprimate, the long-tailed macaque (*M. fascicularis*). These sites are commonly referred to as “monkey forests.” Some monkey forests have little infrastructure and provide no formal provisioning for local macaques [7]. The macaques at these sites may be semi-habituated and avoid humans. In contrast, other monkey forests have created formalized systems for managing their macaque populations that include overseeing the relationships between the macaques, local people, and tourists. The Ubud Monkey Forest has developed a comprehensive staff to manage human–macaque interactions. This includes an onsite medical clinic for humans and a veterinary clinic to address any medical requirements for injured or sick macaques.

**Figure 1:**
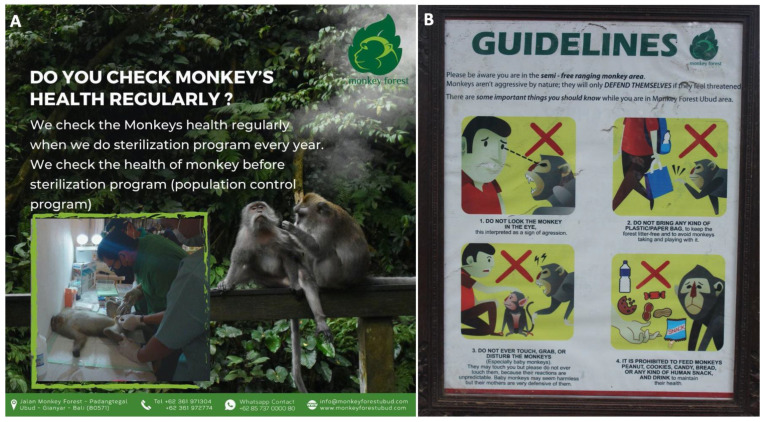
Social media posts and signs developed by the Ubud Monkey Forest. (**A**) Social media post informing the public of their commitment to the health of the long-tailed macaques (*M. fascicularis*) at the site; (**B**) Guidelines sign posted in the forest informing visitors how to behave around the macaques and lower the likelihood of an aggressive interaction.

Sick or injured macaques at the Ubud Monkey Forest are often captured and transported to the veterinary clinic where they are treated. The veterinary clinic includes one full-time staff member who is assisted by the other guards if a wounded or sick macaque requires care. The veterinary clinic primarily treats macaques who have sustained naturally occurring injuries or sicknesses. These can include wounds from fighting, falling from trees, or tumors. The clinic also treats macaques who have sustained injuries from living in a highly populated urban center, namely accidents with cars and motor scooters, electrocutions, or removing plastic bottle caps from cheek pouches. Local veterinarians visit the Ubud Monkey Forest veterinary clinic to address these injuries and may conduct surgeries or administer medication to the macaques. These practices also may assuage the concerns of tourists who visit the site and are worried about injured macaques. Moreover, the Ubud Monkey Forest management communicates to the public their commitment to the health of the macaques using social media platforms and posting signs at the site (Figure 1). Since the veterinary clinic is located at the site, visitors to the Ubud Monkey Forest can see how the sick or injured individuals are cared for, and fully appreciate the commitment of the monkey forest staff to the health of the macaques.

This project highlights the value of the veterinary clinic at the Ubud Monkey Forest. To our knowledge no other on-site veterinary clinic exists at any site where humans and macaques regularly interact. We present data on the type of injuries that macaques face living in a monkey forest situated in an urban environment which is visited by thousands of tourists each day. The data we present demonstrate the Ubud Monkey Forest veterinary clinic’s commitment to monitoring the health of the macaques at the site. This care simultaneously addresses the needs of the macaques, reduces risks associated with injured animals, and reduces anxiety from tourists with concerns for sick or injured animals.

The clinic provides a model for other monkey forests in Bali (and beyond) that may be considering implementing programs that care for local populations of alloprimates. This clinic’s work aligns well with One Health principles and approaches that seek to maximize the viability of ecosystems through monitoring the health of sympatric microbial, floral, faunal, and human communities [8]. The One Health framework posits that human health is linked to healthy ecosystems that include diverse communities of microbes, plants, fungi, and invertebrate and vertebrate animals. Successful One Health programs include projects with durable infrastructures and economic and political stability. This work also demonstrates the importance of incorporating cultural approaches as well as the indigenous knowledge of local stakeholders into programs for sustainably managing their populations of alloprimates [9], and more broadly the populations of wildlife that humans live among [10]. The Ubud Monkey Forest management and staff are an exemplary example of how a small, local village proactively prepares for the challenges presented by exogenous tourism. The overarching objective of this study is to analyze the patterns of injury vs. mortality of a population of urban-dwelling long-tailed macaques with the hope that these data are useful for both researchers and local stakeholders who manage or share their landscapes with sympatric wildlife. More specifically we aimed at understanding the relative odds of injury vs. death by social group, year, age class, and sex among the macaques brought to the clinic. We also aimed to understand the anthropogenic or natural causes of injury vs. death, and the location of injuries and wounds sustained by the macaques inhabiting the site.

## 2. Materials and Methods

### 2.1. Study Site and Study Subjects

The Ubud Monkey Forest (8.519 S, 115.258 E) is located in the village of Padangtegal, which neighbors the larger village of Ubud. The Ubud Monkey Forest is an ~800-year-old temple complex situated on 20.5 hectares of tropical rainforest that includes ~200 plant species [11]. There are three primary temples at the site, the *Pura Dalem Agung* (Great Temple), the *Pura Beji* (Bathing Temple), and the *Pura Prajapati* which lies adjacent to an active cemetery at the site [12]. The Ubud Monkey Forest is among the most visited tourist sites in Bali. Typically, tens of thousands of tourists from more than 70 countries visit the site each year to interact with the macaques [12]. During the study period, the Ubud Monkey Forest was visited by ~1500 paying tourists each day. The funds generated by the entrance fees are used to fund local banks, medical clinics, provide educational opportunities, temple and forest upkeep, and the salaries of the ~100 employees at the site.

During this study, the Ubud Monkey Forest population of long-tailed macaques (*M. fascicularis*) at the site increased (Table 1). Macaque group sizes fluctuated year to year due primarily to new births and mortalities. However, subadult males sometimes emigrated to neighboring groups at the site. Very few males immigrate into the Ubud Monkey Forest population given that the site is situated in an urban landscape that is further surrounded by sawah fields.

The macaques resided in six social groups (Cemetery, Central, East, Michelin, New Forest, Temple) whose core areas were located within the temple forest complex [13]. Some macaques which resided in social groups that were located at the periphery of site (Cemetery, Central, and New Forest) ranged outside the forest temple grounds more frequently than those macaques in centrally located social groups. However, macaques from all the social groups did in fact move outside of borders of the site where they may have crossed roads and/or power lines to forage in fields or gardens, or enter restaurants, shops, or hotels situated near the forest. All the macaques at the site are fully habituated and have spent their entire lives in the presence of humans. The macaques at the site are provisioned by the temple guards. Each day the macaques are provided sweet potatoes but are also given bananas, papaya leaves, corn, and seasonally available fruits and vegetables.

Over the past two decades, the village of Padangtegal has created a management team and staff who maintain and monitor the health of the macaques. This includes a conservation team that cleans the site, composts organic matter, removes and recycles plastic, controls erosion, and plants trees. The site also includes ~40 temple guards who provision the macaques and observe human–macaque interactions and de-escalate agonism between visitors and macaques. The guards also document physical injuries of the macaques or note changes in their behavior that could be associated with injury or illness [12]. Depending on their needs, these animals are brought to the onsite veterinary clinic.

### 2.2. Ubud Monkey Forest Veterinary Clinic

**Figure 2:**
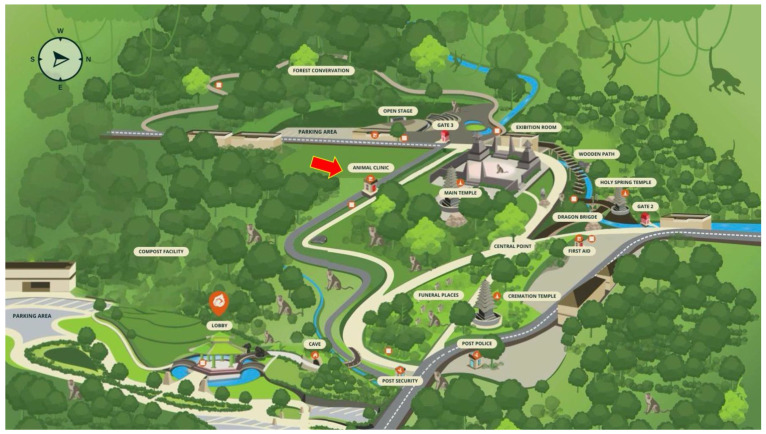
Map of the Ubud Monkey Forest. The red arrow shows the location of the Ubud Monkey Forest Veterinary Clinic which is labelled “animal clinic” on the map.

Unique to the Ubud Monkey Forest is an on-site veterinary clinic that cares for sick and wounded macaques at the site (Figure 2). The clinic was created in 2015 and is fully funded by entrance fees that tourists pay to enter the forest. The clinic is staffed by one full-time employee who works with the temple guards and has local veterinarians on call. The clinic also works closely with the faculties of Veterinary Science and Animal Husbandry at the University of Udayana. The veterinary clinic at the forest is stocked with medicines and antibiotics for sick or injured macaques and blowguns with tranquilizer darts to isolate macaques requiring care. The clinic also includes a surgical table, surgical instruments, refrigerators and freezers for preserving tissues and medicines, medical consumables (i.e., antiseptics, adhesive tape, bandages, gauze, gloves, masks, over the counter medicines, stitches, sutures, syringes), and sterilization supplies. Finally, several isolation cages ensure the safe recovery of the macaques who are to be returned to their social group after treatment. This clinic provides the opportunity for tourists to see the ongoing care available to the macaques.

### 2.3. Data Collection

The data presented in this study were collected by the veterinary clinic at the Ubud Monkey Forest during the course of treatment. Injured macaques were anesthetized, captured, and transported to the veterinary clinic where they were observed and treated by local veterinarians. Deceased macaques were transported to the veterinary clinic where a necropsy was occasionally conducted. All incidences of injury or mortality were documented by the veterinary staff and recorded into a spreadsheet. When possible, the staff noted the date of the incidence, the estimated age based on body size and dental eruption and dental wear, sex, weight of the macaque, group membership of the macaque, a diagnosis of the injury or death, the location of injury (or injuries) on the body, and notes on any treatment that the macaque received. It is important to note that injured animals are difficult to capture. The macaques at the site can identify the blow guns that are used to anesthetize injured individuals and, once a macaque has seen a blow gun, they subsequently flee and vocalize alarm calls to alert all macaques at the site. As a result, capturing injured or sick macaques becomes difficult or impossible. All macaques seen by the veterinary clinic were either those that have been injured and captured or had died and been brought to the clinic resulting in a binarized dataset.

### 2.4. Data Analysis

The purpose of the statistical analyses in the present study are to describe the relative odds of injury vs. death by age class, sex, social group, year, and the cause of injury among the cases brought to the veterinary clinic. Given the binary nature of the data, we fit a multilevel logistic regression model to infer the relationship between injury vs. death and several population- and group-level terms described below. Multilevel models (MLMs) allow for a flexible modeling approach that captures dependence and variability across different levels of the dataset such as study year and social group [14,15].

#### 2.4.1. Model Definition

Let $y_{i}$ represent the binary category for the reason of the clinic visit where 0 = injured and 1 = death for *subject i* (i=1, … , N), where N=275 veterinary cases. We assume $y_{i}\;\sim \;Bernoulli(\theta_{i})$, where $\theta_{i}=P\left(y_{i}=1\right)$ denotes the probability of the veterinary clinic seeing a macaque who has died. In general, we can express θ as some function $f\left(\theta \right)=X\beta +Zb+\epsilon$, where X is an *n*-by-*p* design matrix of population-level effects, β is a *p*-by-1 vector of parameters associated with the population-level effects, Z is an *n*-by-*q* group-level effect design matrix, b is a *q*-by-1 vector of parameters associated with the group-level effects, and ϵ is an error term. We borrow the terms *population-level* and *group-level* effects from Bürkner [14], here population effects are those assumed to be the same across each observation, while group effects are those assumed to vary across grouping variables (i.e., year or social group). Given the constraints placed upon the data such that θ∈[0,1], we fit a generalized linear model (GLM) with the logit link function where $logit\left(\theta_{i}\right)=log\frac{\theta_{i}}{1-\theta_{i}}$, or the log odds of the clinic seeing a macaque who has died [16]. Solving for $\theta_{i}$ and replacing with the function described above, we define the probability of death as $\theta_{i}=\frac{e^{X\beta +Zb+\epsilon }}{1+e^{X\beta +Zb+\epsilon }}$. The interpretation of the results will follow the traditional practice of exponentiating the right-hand side of the equation $\left(e^{\theta }\right)$ to return the odds (odds ratio, OR) instead of the log odds. A value above 1 assumes an increase in the odds of dying, while a value below 1 assumes a decrease in the odds of dying. Note, this also presumes the opposite is true in so much as, given the nature of the data collection, a decrease in the odds of death equates to an increase in the odds of injury (the inverse OR, $\frac{1}{OR}$). Both the OR and inverse OR will be presented.

#### 2.4.2. Bayesian Sampling and Model Selection

We fit a series of multilevel logistic regression models within the R programming environment (R Core Team, version 4.2.3, 2023 [17]) and the *brms* package [15,18]. Bayesian analyses have several benefits over traditional frequentist approaches including a principled approach to uncertainty and the incorporation of prior knowledge for a more theory-guided statistical approach. All associated code is available at https://github.com/ChristopherAWolfe accessible on 6 October 2023. Three total models are fit to estimate the most appropriate suite of population- and/or group-level effects that can predict the types of cases seen by the veterinary clinic. This includes:

1. A baseline intercept only model: $f\left(\theta \right)=\beta_{0}+\varepsilon$;
2. A population-effects only model: $f\left(\theta \right)=\beta_{0}+{X\beta }_{sex}+{X\beta }_{age\;class}+{X\beta }_{group}+X\beta_{year}+{X\beta }_{fighting}+{X\beta }_{anthropogenic/natural}$;
3. A mixed model with population and group effects: $f\left(\theta \right)=X\beta_{p}+Zb_{q}+\epsilon$.

Where $\beta_{p}$ is a parameter associated with each population-level effect above: sex, age class, year, fighting, and anthropogenic/natural, and $b_{q}$ is a vector of parameters associated with the group-level parameters related to social group and year. We use the *loo* package and the expected log pointwise predictive density (elpd) to compare the predictive distributions and overall fit of the models. The model with the largest elpd value is selected for the presentation of all downstream results.

All models assume weakly informative prior distributions: $\beta_{0}\;\sim \;normal(1,1)$, $\beta_{p}\;\sim \;normal(0,1)$, group-level standard deviations sd ~ normal(0,1), and the correlation between group-level effects each year, ρ ~ LKJ(1). Each model is run with the default *brms* settings of 1000 warmup iterations and 1000 sampling iterations across 4 chains, resulting in 4000 total posterior samples per parameter. The adapt delta was set to 0.95 to improve sampling efficiency and provide for a robust model in situations where the posterior geometry may be difficult.

### 2.5. Ethical Considerations

All data analyzed in this study were collected by the Ubud Monkey Forest. The data were collected in adherence to the rules and regulations set forth by the Republic of Indonesia, the Gianyar Regency, and the University of Udayana. In all cases the physical and psychological health of the macaques were prioritized. This study was approved by East Carolina University’s Institutional Animal Care and Use Committee (IACUC—AUP#P094b).

## 3. Results

**Figure 3:**
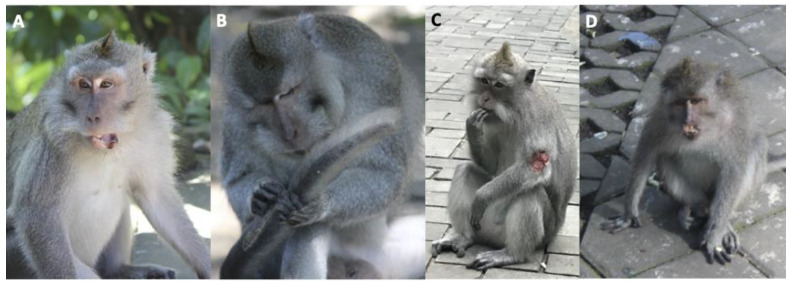
Long-tailed macaque (*M. fascicularis*) wounds at the Ubud Monkey Forest. (**A**) Young adult male with lacerated lower lip from fighting; (**B**) Adult male self-grooming slashed tail wound from fighting; (**C**) Young adult female with open wound on left arm; (**D**) Young adult female with missing upper lip lacerated off from fighting.

**Figure 4:**
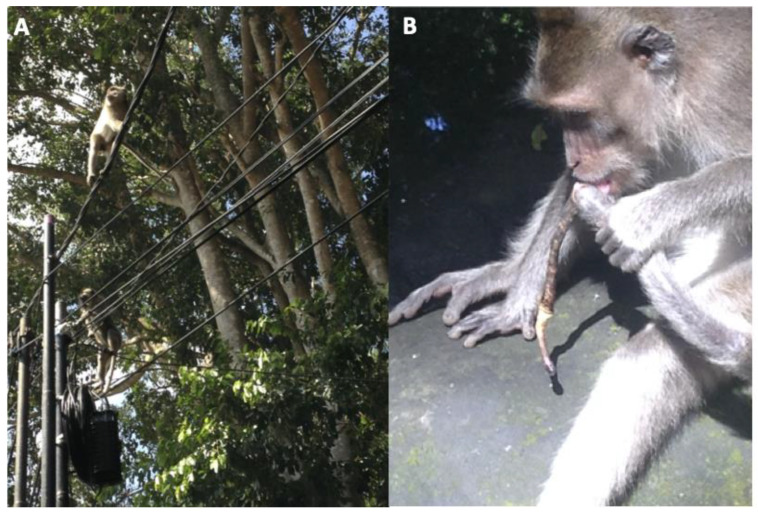
Long-tailed macaques (*M. fascicularis*) inhabiting an urban environment. (**A**) Adult males walking along powerlines situated outside the Ubud Monkey Forest; (**B**) Subadult male licking necrotic tail that was struck by the motor scooter.

**Figure 5:**
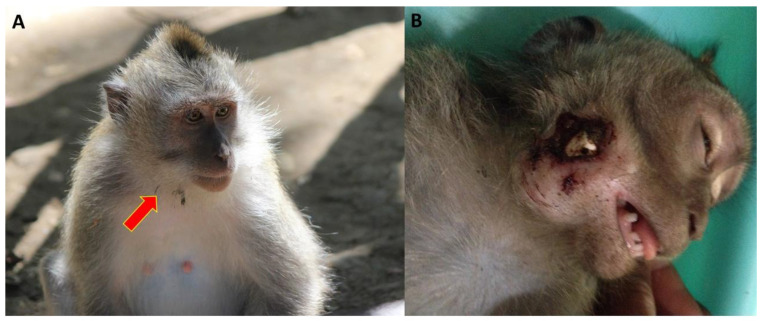
Plastic bottle caps lodged in long-tailed macaque (*M. fascicularis*) cheek pouches. (**A**) Juvenile macaque, red arrow refers to a bottle cap lodged in the cheek pouch; (**B**) Macaque with lodged bottle cap perforating cheek pouch. The bottle cap was surgically removed and treated by the veterinarians at the Ubud Monkey Forest.

In total, the veterinary clinic at the Ubud Monkey Forest recorded 275 events between 2015 and 2018 (Table 2). This included 149 total injuries and 126 total deaths. In a small number of cases, data were collected more than once on the same macaque. This occurred when a macaque was captured, treated, released, and then later re-captured and treated again. Figure 3, Figure 4 and Figure 5 demonstrate the types of injuries observed at the Ubud Veterinary Clinic. The results are presented as follows: model selection criteria, a general summary of the best performing model, a description of the group-level variability across each year, and a description of individual population-level effects.

### 3.1. Model Selection

**Figure 6:**
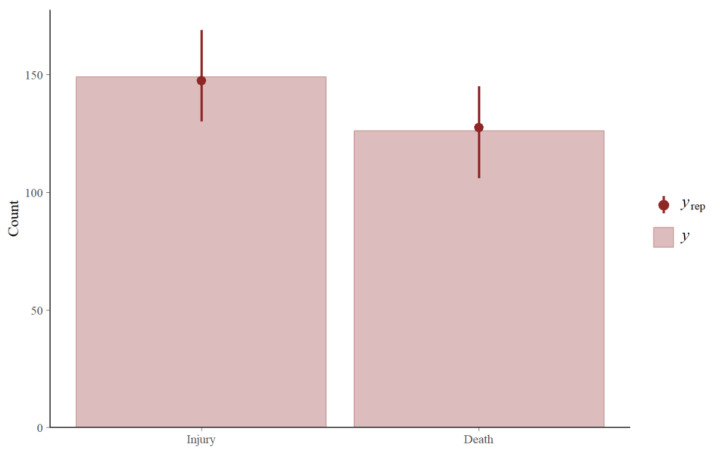
Posterior predictive check $p(\tilde{y}|y)$ showing how well the model predicts an injury vs. death across the sample.

The mixed-effects or multilevel model is the best performing model given the expected log pointwise predictive distribution (elpd). Table 3 presents the results of model selection and includes the elpd value and the standard error of the difference between the best performing model and the remaining two models. The higher the elpd value, the better the performance of the model was. Note that, while the standard error of the difference between the population effect-only model and the multilevel model suggests a small difference in predictive ability, there are several high pareto-k values indicative of model misspecification in the population effect-only model. For this reason and for the higher elpd value, the multilevel model is chosen to complete the remainder of the analyses and results presented below. Figure 6 provides an example of the fit of the model based on the posterior predictive distribution. Given the overlap with the 95% highest posterior density and the raw observations from the dataset, we can conclude the multilevel model recovers the true probability of a macaque found injured or dead among the individuals brought to the clinic.

### 3.2. General Summary of the Multilevel Model

Table 4 presents the brms output of the best performing model. Note, all coefficients are on the log scale and all correlations fall along the interval [−1, 1]. The Estimate is the mean of the posterior distribution for each parameter, the Estimated Error is the standard deviation of the posterior distribution, and L-95 and U-95 are the 95% CI of the posterior. Interpretation is done in comparison to the reference category for each predictor term and relates to the log odds of being found dead and seen by the veterinary clinic. For example, the Estimate for sex is −0.67 where this field is coded a 0 = female and 1 = male. As a result, males have reduced log odds of death as compared to females. However, given the opposite category, this suggests males have increased log odds of injury as compared to females. In general, Table 4 suggests that log odds of death are highest for the youngest macaques (0–1 years old), female macaques, those hurt not fighting, those hurt by anthropogenic causes, and those found in 2017.

The group-level terms have an additional parameter attached known as sd(…) and corr(…). The standard deviation (sd) measures the amount of variation across each social group for each year. The higher the value, the more variability (or difference) between injury vs. death in social groups. The correlation term (corr) measures the relationship across years—i.e., does an increased death rate in one year equate to an increased rate in another or vice versa. In general, all correlation parameters are approximately 0 indicating no year-over-year relationship between injury vs. death. Further, sd is highest in 2017, suggesting increased group-level variability in 2017 as compared to the other groups and years. Both population-level coefficients and group-level variability will be explored further below.

### 3.3. Group-Level Results

**Figure 7:**
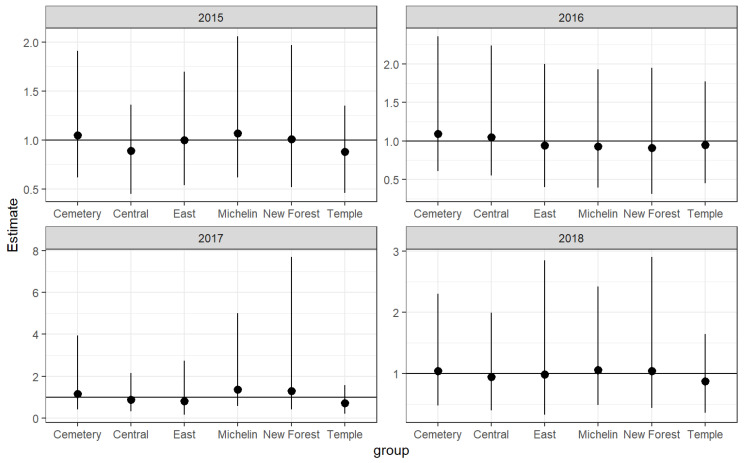
A point range plot demonstrating the estimate and 95% CI of the odds of death at each social group across all four study years. The line at 1 is baseline and assumes equal odds of death vs. injury.

While Table 4 above describes the overall variability present across social groups each year, it does not provide an in-depth summary of the odds of death vs. injury for each group. Figure 7 visualizes the odds of being found dead for each group (odds ratio). These values are obtained by either exponentiating each associated random effect parameter or taking the inverse (1/exp). A line is drawn at 1, which assumes equal odds. Anything above the line suggests an increase in injury vs. death and anything below suggests a decrease. In general, there is a small degree of variability in the odds of injury vs. death across each social group and across each year. The odds of death were highest in 2017 for the Michelin, New Forest, and Cemetery groups whereas the highest odds of injury were in 2017 for the Temple, East, and Central groups. The Temple group saw the highest odds of injury across all 4 years. While the discrepancy year-after-year is rather small, the results do suggest that social group (and resulting proximity to human interaction) does play an outsized role in injury vs. death.

### 3.4. Population-Level Effects

Table 5 presents a summary of results for each of the population-level effects. This includes both the odds of death and/or the odds of injury according to each effect. As it relates to age class, the odds of death were highest for 0- to 1-year-olds, while the odds of injury were highest for 10- to 15-year-olds. Macaques fighting had higher odds of being injured as compared to dying and macaques subject to anthropogenic causes were more likely to die as compared to being injured by natural causes. Males were more likely to be injured, while females were more likely to die. The population level associated with yearly effects indicates 2017 saw the highest odds of death, while 2016 saw the highest odds of injury. Overall, the results indicate that certain population-level effects may influence the probability of dying or being injured at the Ubud Monkey Forest.

### 3.5. Summary Statistics

In Table 6, Table 7, Table 8, Table 9, Table 10, Table 11, Table 12 and Table 13 we present injury and mortality data collected by the Ubud Veterinary Clinic. Across the four years, males accounted for 70.2% (n = 193) of all records and for each individual year males were treated more often than females (Table 2). Males accounted for 64.3% of mortalities across the study period. During the four-year period of this study, the frequency of male mortalities (n = 81) was higher than all the incidences (injuries + mortalities) recorded among the females in this population (n = 80).

The veterinary clinic also estimated the age of the macaques that were examined. For the purposes of simplification, we placed each macaque into one of five age categories as presented in Table 6. Macaques in the 5 to 9 years of age category represented the highest frequency of injuries (n = 53) and mortalities were most frequent for macaques in the 1–4 years of age category (n = 41). We recorded 33 instances of infant mortality. Data on infant mortality rates among free-ranging primates are difficult to document and most infant deaths are attributed to disease and congenital birth defects, and predation.

During the study period there were six social groups (Table 7). Over the study period the total frequency of injuries was different for the six groups. Of the six groups, the New Forest macaques represented the lowest frequency of injuries and mortalities (n = 9), but it was also among the smallest group. By contrast, the neighboring Temple group exhibited the highest frequency of injuries and mortalities (n = 106). The high frequency of injuries exhibited by the Temple macaques may be attributed to the large number of monkeys in the group and the presence of a banana stand near the pavilion and across from the Pura Dalem Agung. The women at the banana stand sold bananas to tourists to feed the macaques, driving the elevated levels of agonism (fighting, chases, threat, and displacements). Subsequently, the banana stand was removed in April of 2018. The highest frequency of mortalities among the groups was found among the Temple group (n = 39) followed by the Cemetery group (n = 27).

Table 8 presents the frequency of the anatomical side (if applicable) where the macaques sustained injuries and Table 9 documents the location of injuries. Most injuries occurred on the body (i.e., sides or ventrum). We note that this classification is very general but may be informative for those studying wildlife in urban landscapes. Injuries to one hand (n = 22) or one leg (n = 24) were prevalent. Injuries to the neck, head, and face including sensory organs (i.e., eyes, and mouth) accounted for 39 of the injuries recorded by the veterinary clinic. Entries on Table 8 and Table 9 do not necessarily represent individual macaques. For instance, a single macaque could sustain injuries at multiple locations on their body (e.g., hand and tail).

The Ubud Monkey Forest macaques live in an urban environment. As a result, macaques exhibit species-typical injuries, and those reflecting the risks of living in human-modified landscapes. We compare the frequency of the injuries that we classified as “natural” (species-typical) vs. “anthropogenic” (indirectly or directly caused by humans). The frequencies of natural and anthropogenic injuries are summarized in Table 10.

We further break down the types of natural injuries in Table 11, and in Table 12 we summarize the frequencies of injuries and mortalities associated with fighting. Over the course of the four-year study period, there were more naturally occurring injuries than those that were anthropogenic. Across the study period the frequency of natural injuries fluctuated, peaking in 2016.

Table 11 presents the types naturally occurring injuries and mortalities recorded by the veterinary clinic over the study period. Wounds were over-represented in the dataset. Overall, 124 wounds were recorded by the clinic. Falls from trees and other substrates accounted for 45 of the cases; 84.4% of the falls resulted in death.

Comparatively, very few of the macaque injuries documented by the veterinary clinic could be attributed to fighting (Table 12). In fact, fighting among the Ubud Monkey Forest macaques accounted for 9.4% of all the injuries reported (n = 14). 

In Table 13 we present the frequencies of injuries and mortalities associated with living in an urban habitat as collected by the Ubud Veterinary Clinic for the 2015–2018 study period. These injuries and mortalities were associated with electrocutions, vehicular collisions, gunshots, and plastic pollution. Electrocution from powerlines (Figure 4A) resulted in 20 injuries and mortalities, and 12 macaques were struck by a car or motor scooter (Figure 4B). Unfortunately, all the macaques who were struck by motor scooters or cars or shot by local people died. Five of the reports include macaques who had plastic bottle caps lodged in their cheek pouches, typically these macaques were juveniles (Figure 5A). If left unattended, these bottle caps can result in infected oral wounds (Figure 5B). It is a testament to the veterinary team and forest management that none of these injuries resulted in mortalities.

## 4. Discussion

Navigating in a society can be difficult for any animal living in a social group. These challenges can be compounded by those animal populations inhabiting urban environments. Alloprimates live among humans to varying degrees, with anthropogenic impacts on their health, behavior, and biology that are often difficult to document. The integrative economic, religious, environmental, and health framework developed by the Ubud Monkey Forest is a strong example of One Health Initiatives [8].

Recent studies have documented that several populations of alloprimates are exposed to human impacts in nuanced ways that are sometimes difficult to document, including exposure to human diseases [19,20,21,22], environmental toxins [23,24,25], and the effects of climate change [26]. However, what has been missing are clear insights into the role that sex, age, social group, and anatomical location play in these patterns. Also missing are estimates of natural and anthropogenic causes of injury and mortality. We address this gap by presenting frequency data and then modeling this data for a population of macaques inhabiting an urban environment while illuminating some of challenges alloprimates experience.

### 4.1. Sex

The data presented in this study revealed that the injuries were male biased, however mortalities were female biased. Male macaques possess long, dagger-like canines that they use to slash during fights, sometimes resulting in open wounds that can become infected. The provisioning program at the Ubud Monkey Forest has been successful but has resulted in a high population density [12]. Since the forest is situated in an urban setting, there are very few options for males living outside of the site to immigrate into the Ubud Monkey Forest population, and also few options for the males who inhabit the site to emigrate out, leading to a comparatively high number of males [27], which sometimes results in high intra-male aggression. The over-representation of males with injuries may be linked to higher levels of agonism in intra-male encounters. Sex ratios among provisioned and non-provisioned long-tailed macaque populations vary [28]; at the Ubud Monkey Forest, the ratio of males to females is comparatively high, as are the number of males residing in this population [29].

Although the veterinary clinic documented more total injuries and total mortalities among the males at the forest, the proportion of female mortality was higher than male mortality. The high percentage of mortalities may be due in part to sampling bias by which dead animals are easier to collect and document. As previously noted, injured macaques easily recognize the blow guns and produce alarm calls when they are identified. A single alarm call by an individual macaque usually results in all macaques within earshot fleeing, and/or ascending trees. This makes their capture by the veterinary clinic staff difficult or impossible.

### 4.2. Age

Injuries were most frequent for the 5–9 years of age category. The odds of death were highest for 0- to 1-year-old macaques, while the odds of injury were highest for macaques 10 to 15 years of age. At the Ubud Monkey Forest, male macaques typically reach sexual maturity by age 6, and females by 3.5 years [29]. For males, this is often a period when they begin asserting themselves within their group leading to increased levels of both affiliation and aggression, and potentially transferring to another group [30]. Within the 1–4 years of age category, the veterinary clinic recorded the most mortalities. These deaths were attributed to falls, wounds, sickness, electrocution, and collisions with cars and motor scooters. There were no recorded cases of infanticide, and throughout our >20 years of observing the macaques at Ubud Monkey Forest, we have not documented any cases of infanticide.

### 4.3. Social Groups

The frequency of injuries and mortalities differed each year and among each of the six social groups. The highest frequency of injuries was documented among the Temple Group followed by the Central Group (Table 7). There was some variability in the odds of injury vs. death across the six social groups, across each year. The chances of death were highest for the Michelin, New Forest, and Cemetery groups in 2017. The highest likelihood of injuries was in 2017 for the Temple, East, and Central groups. Across all 4 years of the study, the Temple group experienced the highest odds of injury.

During this study, the Temple and Central groups had banana stands located at their core areas. These stands were set up by local people to sell bananas to tourists who wanted to feed the macaques. Discussions with the temple guards who provision the macaques and observe tourist-macaque interactions daily, note that the removal of these stands has resulted in a reduction in the levels of aggressions between the macaques for bananas. On rare occasions fighting among the macaques resulted in wounds that lead to death. However, most wounded macaques were transported to the veterinary clinic where they were treated (e.g., cleaning superficial wounds, providing antibiotics, surgery) and released once healthy (Buana and Buda, personal observation).

### 4.4. Anatomical Location

The anatomical location of the wounds was also recorded by the veterinary clinic. There were no differences in the anatomical side that a macaque was injured on, and most injuries occurred on the body (Table 8 and Table 9). Aside from the body, most injuries occurred on only one hand, one leg, or the head. These results were surprising given that our team has recorded the locations of wounds on the body of the macaques at the site for >20 years. We have noted several wounds on the tails of the macaques demonstrating our recording biases in previous studies. A bias towards recording tail wounds may be attributed to the fact that tail wounds are easy to observe given their length and are characterized with comparatively little hair and shorter hairs which reveal wounds more readily. Moreover, macaques frequently self-groom their wounded tails as depicted in Figure 3B and Figure 4B which draws attention to those wounds by the primatologist(s) observing them.

### 4.5. Natural Injuries and Mortality

We categorized all injuries and mortalities as “natural” or “anthropogenic” in origin. However, some cases made it challenging to determine if a condition was entirely from a natural or anthropogenic source. For instance, a fall may be due to natural causes (e.g., being chased, broken branch) but becomes fatal after landing on asphalt or concrete. As a result, this death could be classified as anthropogenic. We chose to classify it as natural due to the origin (fall). However it demonstrates the intersection of the forest–urban environment.

Overall, there were more naturally occurring injuries (n = 135) and mortalities (n = 99) than incidences of anthropogenic origins (injuries: n = 14, mortalities: n = 27). Injuries and mortalities amongst these macaques were most frequently attributed to falling from trees and macaque-induced wounds. Although these data may seem surprising given that catarrhine monkeys utilize trees (and other substrates) to sleep, rest, and avoid conspecifics and predation and would be expected to have greater safety in these spaces. However, their substantial use of these substrates and increased risk of injury and death due to velocity makes these outcomes more likely. Interestingly, primatologists have rarely noted falls among nonhuman primates. However, Goodall documented 51 falls from chimpanzees (*Pan troglodytes*) spanning over two years at Gombe [31]. The paucity of documented falls among free-ranging nonhuman primates may be due to the challenges of recording this event in the wild. The fact that we have such clear numbers is another insight into the benefits of the monkey forest management and veterinary clinic.

The veterinary clinic also documented and treated multiple macaque-induced wounds associated with fighting. The majority of the macaques who sustained wounds survived due to the care of the veterinary team which cleaned, sutured, stitched, and medicated the macaques (Buana and Buda, personal observation). In addition, the care team was able to provide protected space for the macaques to regain their health while healing. This additional care probably helped ensure proper healing, and mitigated loss of function.

### 4.6. Anthropogenic Injuries and Mortality

There were 14 injuries and 27 mortalities attributed to anthropogenic causes (Table 13). It is interesting that although these macaques frequently range outside the protected forest temple complex, this rarely resulted in injuries and mortalities linked to living in an urban environment. Those that did occur were associated with electrocutions, vehicular collisions, gunshots, and plastic pollution.

### 4.7. Electrocutions

Electrocutions were a major cause of anthropogenic mortality in this population of macaques. Death associated with electrocution is not uncommon in monkeys inhabiting anthropogenically disturbed habitats. For instance, cause of death was attributed to electrocution in 73% of the deaths among four species of African monkeys in Diana, Kenya [32]. This is higher than the 55% recorded in the Ubud Monkey Forest. This variation in outcomes could be caused by differences in habitat structure (e.g., how much of their home range is bordered by powerlines), and the exposure to unsafe electrical facilities. In Ubud, the majority of the macaques who were electrocuted resided in groups whose range bordered busy roads (Cemetery Group and Central Group). These groups frequently walk along the power lines that border the roads to avoid motorized vehicles (Figure 4A). The majority of macaques who died following electrocution had made physical contact with a raised substation on the border of the forest. After this risk was identified by the veterinary clinic and forest management, it was removed by the electric company.

### 4.8. Vehicular Collisions

The Ubud Monkey Forest is bordered on northern, western, and southern sides by moderate to heavy traffic. These areas provide crucial thoroughfares for locals, taxis, and tourists, many of whom are traveling to work at or visiting the forest. Heavy traffic combined with ever expanding macaque populations results in an environmental mismatch and potential hazards. During this study, 12 macaques were struck by motor scooters or cars and subsequently died. There are similar patterns in nonhuman primates in other shared urban landscapes. For instance, vehicular collisions were the most common source of injury and mortality in four species of monkeys in Diana Kenya [33]. In comparison, vehicular accidents represent the most common source of anthropogenically originating mortality in the Ubud population. However, electrocutions were the leading reason for injury and the second most frequent source for mortalities.

These risks demonstrate the persistent threat of converting nonhuman primate habitats for human purposes. However, the local community’s response to these threats speaks to the excellent communication and cooperation between the management of the Ubud Monkey Forest, the veterinary team, and the adjoining villages. In recognition of this growing challenge, the monkey forest staff coordinated with village leaders and local community members to change the traffic routes surrounding the forest to decongest the area surrounding the forest, and reduce the vehicular risks to the macaques.

### 4.9. Gunshots

The third leading anthropogenic cause of death in this study were gunshot wounds. It is not surprising that all that macaques who died from gunshot wounds were members of the Temple Group whose home range borders against mixed-use land. Members of this group sometimes raid the gardens of people living near the forest. In addition, they become emboldened by permissive tourists who provide treats to the macaques who enter local homestays, hotels, shops, and restaurants situated near the forest. Although the use of firearms in Bali is rare, it is unfortunately one of the few methods landowners have to address destructive crop raiding macaques. In general, hunting is forbidden in Bali. However, reports of the Balinese macaques shot by local people have been documented in at least four locations in Bali where the two primate species interact [7,34]. As the macaque population has grown, the Temple Group has further dispersed into these human-dominated spaces. Recognizing the detrimental role of population growth in group expansion, the Ubud Monkey Forest in partnership with the University of Liège (Belgium) and the University of Udayana (Bali) have initiated a sterilization program [35,36].

### 4.10. Plastic Pollution

A final but important category of injuries was plastic pollution. Over the course of this study, the veterinary clinic treated four macaques with plastic bottle caps lodged in their cheek pouches (Figure 5A,B). Plastic pollution is an ongoing hazard in Bali [37], and inquisitive younger macaques will place bottle caps in their cheek pouches and have difficulty removing them. On rare occasions, these bottle caps may perforate their cheeks and result in open wounds which increases the potential for infection. As a result, the macaques were sedated, and the veterinary team removed the bottle cap. The Ubud Monkey Forest is actively addressing the problems of plastic pollution by prohibiting tourists from entering the forest with any plastic bottles with removable lids. In addition, they developed a conservation program aimed at removing all plastic from the site [12]. This program highlights the Ubud Monkey Forest’s proactive approach to reducing plastic pollution which is a growing concern that many populations of nonhuman primates face [25].

### 4.11. The Ubud Monkey Forest Veterinary Clinic as a Model of Culturally Integrated Animal Care

This clinic addresses the health of these macaques as part of the forest leadership’s overall management strategy. The primary focus of the veterinary clinic is to ensure the best quality of health for the macaques who inhabit the site and to analyze the patterns of injuries and mortalities to develop programs to reduce or prevent them in the future. The success of the veterinary clinic emerges from the Ubud Monkey Forest’s commitment to creating an environment that maximizes the physical and psychological health of the macaques, while addressing tourist concerns of injured animals. Inadvertently, it has created a robust and successful One Health framework.

This forest has developed a comprehensive system for balancing the needs of the local Balinese people, the macaques who inhabit the site, and the tourists who visit. The monkey forest is considered as a sacred site by the Balinese people, is an active site for ceremonies, rituals, and offerings to the gods, and is attended by locals to participate in important religious and calendric celebrations. At Ubud, and among other populations found throughout southeast Asia, the macaques at this site are associated with the monkey god *Hanuman*, who is also conceptualized as a hero and plays a primary role in a central Hindu narrative, *The Ramayana* [38].

The macaques at the site are also interwoven into the Balinese philosophy of *Tri Hita Karana* which posits that health, happiness, and prosperity are achieved by living harmoniously with God, the spiritual world, and the environment [39,40]. This cultural, religious, and philosophical approach is thought to lead towards a prosperous life with respect for (nonhuman and human) others [41]. In this religious model, supporting the macaques would bolster harmonious living with the environment, thus having direct benefits for humans. In addition to Tri Hita Karana being used as an explanation for veterinary care, it also supports the practices of provisioning the macaques at the sites directly through feeding locations and indirectly through temple offerings.

Although other monkey forests in Bali are managed by Hindus who presumably embrace Tri Hita Karana, the level this framework is extended to the macaques in Ubud is unprecedented. This difference is likely due to the extensive economic benefits associated with tourism at this site. Entrance fees into the site supports community schools and health centers while providing religious and animal husbandry support in the forest temple complex. There is a clear relationship where the humans support the macaques, and the macaques generate revenue which in turn supports the humans.

Veterinary care plays an additional role in this economic, religious, and One Health model. The clinic addresses the worried concerns of tourists who visit the site who are anxious about injured macaques. Having the clinic on view for visitors and actively addressing these concerns helps reinforce the public face of the forest as a sanctuary for macaques and tourists alike. In addition, treating injured, sick, and stressed animals may reduce the chances of a distressed macaque biting a human. Although macaques at this site do not harbor rabies, reducing macaque inflicted wounds on humans also reduces the chances of transmitting zoonotic diseases. Finally, the thoughtful use of educational signs throughout the forest and on social media communicates the management’s commitment to the health and well-being of the macaques (Figure 1). These signs also elucidate steps that could be taken by visitors to support the macaques’ health (e.g., keeping all bottles with caps safely in their bags and appropriately disposing of trash).

The Ubud Monkey Forest continues its long tradition of proactively addressing local endogenous and global exogenous challenges in an integrative One Health framework. Whether it be modifying exposed electrical substations, changing traffic patterns, or redirecting groups away from heavily modified urban spaces, insights from the veterinary team have enabled management to further support the forest’s goals. Data from this clinic also provide important insights into alloprimate health in a mixed-use, urban forest. Taken together, this clinic provides an excellent model that could be scaled up or closely replicated by other monkey forests across Indonesia, southeast Asia, and in regions where humans and alloprimates are interconnected.

## 5. Conclusions

The Ubud Monkey Forest in Bali, Indonesia has developed an impressive model of integrative care using the One Health framework that simultaneously supports the needs of local humans, resident macaques, and tourists. Entrance fees to this forest provide funding for medical care as well as enhanced educational and economic opportunities for locals. These funds have also been thoughtfully reinvested into the forest through land purchases, reforestation, and initiatives to reduce soil erosion and plastic pollution [12]. Although Ubud is not the only monkey forest in Bali it stands out as a leader in managing human–macaque interconnections. These free-ranging macaques are provisioned and protected and are the only population that we know of that has a veterinary clinic on the premises. The insights stemming from the veterinary team have already been particularly important to the monkey forest management in developing long-term management plans with their community leadership.

This study demonstrates some of the obstacles that long-tailed macaques (*M. fascicularis*) face living in a highly populated urban center. We present data on the frequency of injuries and mortalities collected by the veterinary clinic at the Ubud Monkey Forest over a four-year period (2015–2018). The record keeping of the Ubud Monkey Forest veterinary clinic enabled our collaborative team to assess the frequency and causes of injuries and mortalities and generate data-driven models. These data are particularly interesting given the growing number of alloprimates living in ecosystems modified by humans.

The data presented in this study are uncommon and have revealed patterns of injury and mortality for a highly social alloprimate that are rarely documented to this degree. However, future studies at the Ubud Monkey Forest examining the rates of injury and death relating to the actual age and sex distributions of the population and rates of recovery and successful reintroduction due to veterinary care are warranted and would provide a better understanding of the challenges faced by long-tailed macaques and other species of gregarious alloprimates. The result of this work will hopefully be helpful in implementing similar programs at other sites to address the social and anthropogenic challenges that alloprimates face and that may result in injury. These data are also useful for wildlife management teams inside and outside of Bali. For instance, these data will help primatologists and other wildlife experts better understand the relationships of injury and mortality with different sex and age classes, social groups, and anatomical locations and the role of natural and anthropogenic injuries and mortalities on alloprimate populations. Given that in 2018, there were ~800 macaques at the site, the number of injuries and mortalities at the Ubud Monkey Forest is low and demonstrates the efficacy of the veterinary clinic and the commitment by the forest management to the health and well-being of the macaques at the site [12].

## Figures and Tables

**Table 1:** Group sizes for each long-tailed macaque (*M. fascicularis*) study group during the 2015–2018 study period at the Ubud Monkey Forest.

Social Group	2015	2016	2017	2018
Cemetery	85	78	96	92
Central	152	146	148	199
East	90	113	138	104
Michelin	85	108	104	152
New Forest	84	109	102	105
Temple	163	124	161	112
Total	659	678	749	764

Population census data were collected by the Ubud Monkey Forest and Brotcorne [13].

**Table 2:** The frequency of long-tailed macaque (*M. fascicularis*) injuries and mortalities for females and males by year (2015–2018) at the Ubud Monkey Forest.

**Females**			
**Year**	**Injuries**	**Mortalities**	**Total**
2015	4	8	12
2016	19	18	37
2017	4	10	14
2018	8	9	17
Total	35	45	80
**Males**			
**Year**	**Injuries**	**Mortalities**	**Total**
2015	26	19	45
2016	43	24	67
2017	22	20	42
2018	21	18	39
Total	112	81	193

For two incidences, the sex of the macaque was not recorded.

**Table 3:** Model selection results from the loo package listed in descending order from best to worst performing model.

Model	ELPD	ELPD Difference	Standard Error of the Difference
Multilevel	−176.0	0.0	0.0
Population-Effects Only	−180.8	−4.8	4.2
Intercept Only	−190.6	−14.6	6.0

**Table 4:** General summary of the best fitting model. The Estimate is the mean of each parameter, Estimated Error is the standard deviation, and L-95 and U-95 are the upper and lower limits of the 95% CI. Note, the sd() parameters and the population-level terms are on the log scale.

**Group-Level Terms**				
**Parameter**	**Estimate**	**Estimated Error**	**L-95**	**U-95**
sd(2015)	0.28	0.24	0.01	0.92
sd(2016)	0.35	0.30	0.01	1.07
sd(2017)	0.62	0.44	0.03	1.67
sd(2018)	0.41	0.34	0.02	1.23
cor(2015,2016)	−0.07	0.45	−0.86	0.78
cor(2015,2017)	0.01	0.44	−0.81	0.81
cor(2016,2017)	0.02	0.44	−0.79	0.81
cor(2015,2018)	−0.02	0.45	−0.83	0.81
cor(2016,2018)	0.03	0.46	−0.81	0.82
cor(2017,2018)	0.08	0.44	−0.77	0.85
**Population-Level Terms**				
**Parameter**	**Estimate**	**Estimated Error**	**L-95**	**U-95**
βIntercept	1.60	0.49	0.68	2.61
β1–4 years old	−0.80	0.38	−1.55	−0.08
β5–9 years old	−1.34	0.38	−2.09	−0.61
β10–15 years old	−1.76	0.40	−2.56	−0.97
β16+ years old	−0.48	0.77	−2.00	1.06
βsex_male	−0.67	0.28	−1.21	−0.12
βfighting	−0.75	0.55	−1.82	0.30
βanthropogenic	0.76	0.37	0.05	1.49
β2016	−0.52	0.40	−1.28	0.32
β2017	0.17	0.49	−0.75	1.17
β2018	−0.21	0.45	−1.07	0.71

Intercept = 0–1 years old, female, not fighting, natural, 2015.

**Table 5:** A summary table indicating the mean posterior odds of a macaque dying and/or being injured according to each population-level effect. Values above 1 assume increased odds and values below 1 assume decreased odds.

Parameter	Odds of Death	Odds of Injury
βIntercept	5.01	0.20
β1–4 years old	0.45	2.24
β5–9 years old	0.26	3.84
β10–15 years old	0.17	5.88
β16+ years old	0.61	1.64
βsex_male	0.51	1.95
βfighting	0.47	2.13
βanthropogenic	2.15	0.47
β2016	0.59	1.70
β2017	1.17	0.85
β2018	0.81	1.24

Intercept = 0–1 years old, female, not fighting, natural, 2015.

**Table 6:** The frequency of long-tailed macaque (*M. fascicularis*) injuries and mortalities based on age categories throughout the study period by year (2015–2018) at the Ubud Monkey Forest.

Age Category	Injuries	Mortalities
0–1 years	8	33
1–4 years	35	41
5–9 years	53	33
10–15 years	51	18
16+ years	2	1
Total	149	126

**Table 7:** Frequencies of injuries and mortalities shown in parentheses for the six social groups of long-tailed macaques (*M. fascicularis*) at the Ubud Sacred Monkey Forest for 2015–2018.

Year	Cemetery	Central	East	Michelin	New Forest	Temple	Total Injuries	Total Mortalities
2015	1(1)	5 (2)	9 (12)	0 (0)	0 (0)	17 (12)	32	27
2016	12 (14)	15 (9)	4 (2)	4 (3)	3 (1)	24 (13)	62	42
2017	2 (4)	7 (8)	1 (0)	4 (9)	0 (2)	12 (7)	26	30
2018	4 (8)	5 (4)	0 (0)	5 (6)	1 (2)	14 (7)	29	27
Total	19 (27)	32 (23)	14 (14)	13 (18)	4 (5)	67 (39)	149	126

**Table 8:** Frequency of injuries and mortalities based on the anatomical side of the Ubud Monkey Forest long-tailed macaques (*M. fascicularis*) for the study (2015–2018). For some records, anatomical side was not documented.

Anatomical Side	Injuries	Mortalities
left	16	4
left & right	3	3
right	25	3
N/A	65	70
Total	109	80

**Table 9:** Frequency of the location of bodily injuries and mortalities of the Ubud Monkey Forest long-tailed macaques (*M. fascicularis*) for the study (2015–2018). For some records, the location of the injury was not documented.

Body Part	Injuries	Mortalities
Abdomen	1	1
Ano-genital	3	3
One arm	1	0
Back	3	0
Body (sides and ventrum)	31	60
Eye	1	0
Face	4	0
Feet (both)	0	1
Foot	5	2
Hand	22	3
Hands (both)	2	1
Head	18	4
Leg	24	2
Legs (both)	0	1
Mouth	14	1
Neck	2	2
Scrotum	2	0
Tail	7	2
Total	140	83

**Table 10:** Frequency of “natural” and “anthropogenic” injuries for the population of long-tailed macaques (*M. fascicularis*) at the Ubud Monkey Forest for 2015–2018.

Year	Natural	Anthropogenic	Total
2015	27	5	32
2016	56	6	62
2017	24	2	26
2018	28	1	29
Total	135	14	149

**Table 11:** Total frequency of each type of naturally occurring injuries and mortalities for the population of long-tailed macaques (*M. fascicularis*) at the Ubud Monkey Forest for 2015–2018.

Diagnosis/Treatment	Injuries	Mortalities
Dead (after birth)	0	5
Dead (no reason recorded)	0	15
Fall	7	38
Fighting	14	4
Operation	1	0
Released	1	0
Seizure	1	1
Sick	3	6
Starvation	0	1
Surgery (recovering after surgery)	2	1
Tumor	7	0
Ulcer	3	0
Wound(s)	96	28
Total	135	99

Wounds refer to injuries resulting in cuts, slashes, or impacts.

**Table 12:** Frequency of injuries from non-fighting and fighting for the population of long-tailed macaques (*M. fascicularis*) at the Ubud Monkey Forest for each year of the study. Mortalities associated with fighting are presented in parentheses.

Year	Non-Fighting Injuries	Fighting Injuries	Total
2015	22	10 (3)	32
2016	58	4 (0)	62
2017	26	0	26
2018	29	0 (1)	29
Total	135	14 (4)	149

**Table 13:** Total frequency of each type of “anthropogenic” injury and mortality for all years (2015–2018) for long-tailed macaques (*M. fascicularis*) at the Ubud Monkey Forest.

Diagnosis	Injuries	Mortalities
Bottle cap (in cheek pouch)	5	0
Electrocution	9	11
Hit by car	0	3
Hit by motor scooter	0	9
Gunshot	0	4
Total	14	27

## Data Availability

All data presented and analyzed in this study were collected by the Ubud Monkey Forest. We are not permitted to share these data. These data are considered private and sensitive by the Ubud Monkey Forest management and the leadership of the village of Padangtegal.
